# Among-population proteomic differences in *Schistocephalus solidus* based on excretory/secretory and total body protein predictions

**DOI:** 10.1186/s13071-025-06807-x

**Published:** 2025-05-20

**Authors:** Anni Wang, Daniel I. Bolnick

**Affiliations:** Department of Ecology and Evolutionary Biology, University of Connecticut, Storrs, CT 06269 USA

**Keywords:** Parasite, Cestode, Proteomics, Secretome, Stickleback, Coevolution

## Abstract

**Background:**

Parasites secrete and excrete a variety of molecules that evolved to help establish and sustain infections within hosts. Parasite adaptation to their host may lead to between-population divergence in these excretory and secretory products (ESPs), but few studies have tested for intraspecific variation in helminth proteomes.

**Methods:**

*Schistocephalus solidus* is a cestode that parasitizes the threespine stickleback, *Gasterosteus aculeatus*. We used an ultra-performance liquid chromatography–mass spectrometry protocol to characterize the ESPs and whole-body proteome of *S. solidus*. Specifically, we characterized the proteome of *S. solidus* at the plerocercoid stage from wild-caught stickleback from three lakes on Vancouver Island (British Columbia, Canada) and one lake in Alaska (USA). We tested for differences in proteome composition among the four populations and specifically between ESPs and body tissue.

**Results:**

Overall, we identified 1362 proteins in the total proteome of *S. solidus*, with 542 of the 1362 proteins detected exclusively in the ESPs. Of the ESP proteins, we found signaling peptides and transmembrane proteins that had not been previously detected or characterized in *S. solidus*. We also found that protein spectrum counts varied greatly among all lake populations.

**Conclusions:**

These population-level differences were observed in both ESP and whole-body tissue types. Our study suggests that *S. solidus* can excrete and secrete a wide range of proteins which are distinct among populations. These differences might reflect plastic responses to host genotype differences, or evolved adaptations by *Schistocephalus* to different local host populations.

**Graphical Abstract:**

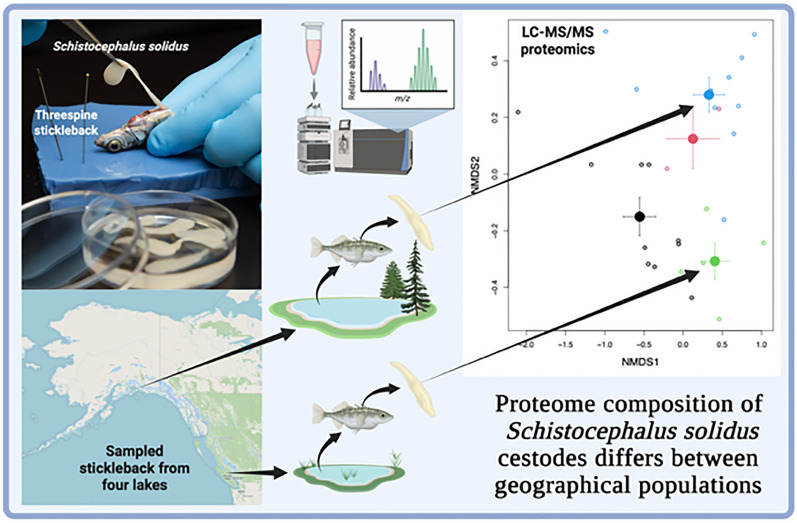

**Supplementary Information:**

The online version contains supplementary material available at 10.1186/s13071-025-06807-x.

## Background

Parasitic worms, or helminths, can manipulate their hosts through various intricate strategies to establish infections, which facilitates long-term survival. Among these strategies, one prominent tactic involves the production and subsequent release of an extensive range of excretory and secretory products (ESPs), which serve as pivotal mediators in the interplay of host–parasite interactions [1]. These molecules can help parasites escape the host immune system, alter host behavior, and modify host physiology [2]. Among helminth parasites, protein ESPs may include proteinase inhibitors, proteinases, heat shock proteins (HSPs), and venom allergen-like proteins [3–5]. Extensive studies on helminth-derived ESP or their protein subset mixtures have demonstrated partial or total protective responses in hosts with these helminth infections [6, 7]. ESPs have been analyzed in parasitic helminths such as *Strongyloides ratti*, *Ascaris suum*, *Trichuris muris*, and *Brugia malayi* [8–11].

Despite the extensive work on helminth manipulation of host traits, some important gaps remain. For instance, intraspecific variation in helminth proteomes has been mostly overlooked, with work focusing on describing species-level proteome composition and function. This oversight is important because helminths engage in a coevolutionary arms race with their hosts [12, 13]. Parasites evolve new immunosuppressive strategies while the hosts develop countermeasures. This coevolution should lead to rapid evolution of both species. When this rapid evolution takes place independently in geographically separate populations [14], we may expect that parasite immune modulation traits will differ among populations. This may lead to among-population divergence in the composition of the parasite proteome. However, intraspecific geographic variation in parasite proteomes has not been investigated. Here, we begin to rectify this research gap, by (1) describing the proteome of a widely studied tapeworm parasite and (2) testing for among-population differences in the proteome.

*Schistocephalus* is a genus of tapeworms within the family Diphyllobothriidae, specializing in parasitizing fish hosts as plerocercoid larvae through the ingestion of parasitized copepods [15]. *Schistocephalus solidus* is recognized for its infection within the threespine stickleback, *Gasterosteus aculeatus*, as it is well documented for studying ecological processes and the genetic architecture of evolution in the wild [16–18]. *Schistocephalus solidus* exhibits a complex life cycle that requires transmission through two intermediate hosts and a final bird host. The first intermediate host is a cyclopoid copepod parasitized by the coracidium stage of *S. solidus*, which hatches from an egg deposited in the water. *Schistocephalus solidus* then develops into its procercoid stage in the copepod gut, which is subsequently ingested by the second intermediate host, *G. aculeatus*. Within the fish, *S. solidus* penetrates the gut wall to enter the peritoneal cavity, where it develops into its plerocercoid stage and grows to reproductive size. If the fish is consumed by a fish-eating bird, the tapeworm rapidly matures into an adult within the host intestine, where it mates (or selves) and produces eggs [78]. Sticklebacks infected at the plerocercoid stage have been documented to have an altered immune system, morphology, and behavior, making the plerocercoid an appealing stage for studying potential ESPs responsible for these changes [19–21].

Initial proteomic work on the host–parasite system focused on characterizing subsets of proteases and transferases responsible for different developmental stages in *S. solidus* that promote growth and survival [22, 23]. Subsequent proteomic work has focused on characterizing the parasite proteome and ESP at the plerocercoid stage, detection of host proteome changes due to ESPs, and parasite ESPs increasing respiratory burst activity in stickleback [24–26]. While these studies have helped establish a reference proteome and identify potential ESPs interacting with the host proteome, they have largely not considered the possibility that the proteome evolves and thus differs among populations.

Many freshwater populations of stickleback have evolved resistance to *S. solidus* [27]. Some lake populations are more resistant, initiating an extensive peritoneal fibrosis response that suppresses tapeworm growth and viability; other lake populations evolved a tolerance strategy by suppressing fibrosis [28, 29]. Transcriptomic analysis reveals among-population variation in fish immune genes in stickleback fish to *S. solidus* across different lakes [30, 31], which can be induced by injecting *S. solidus* proteins into the fish [32]. However, host fibrosis response also depends on *S. solidus* genotype [33], suggesting that both host and parasite are co-evolving. We hypothesized that this coevolution would lead to divergence in *S. solidus* proteome composition among plerocercoids from diverse lake populations. We further hypothesized that among-population divergence would be greater for excretory/secretory products than for whole-body proteomes, potentially influencing diverse host–parasite interactions [29]. To test these hypotheses, we conducted proteomic profiling of *S. solidus* whole-body tissue and their ESPs sampled from stickleback hosts in four different lakes in British Columbia, Canada and Alaska, USA. Specifically, we identified signaling peptides, excreted proteins, transmembrane proteins, and differences in proteome composition across these different lakes that could be molecules responsible for establishing infection or maintaining host–parasite interactions.

## Methods

### Fish and tapeworm collection

**Fig. 1:**
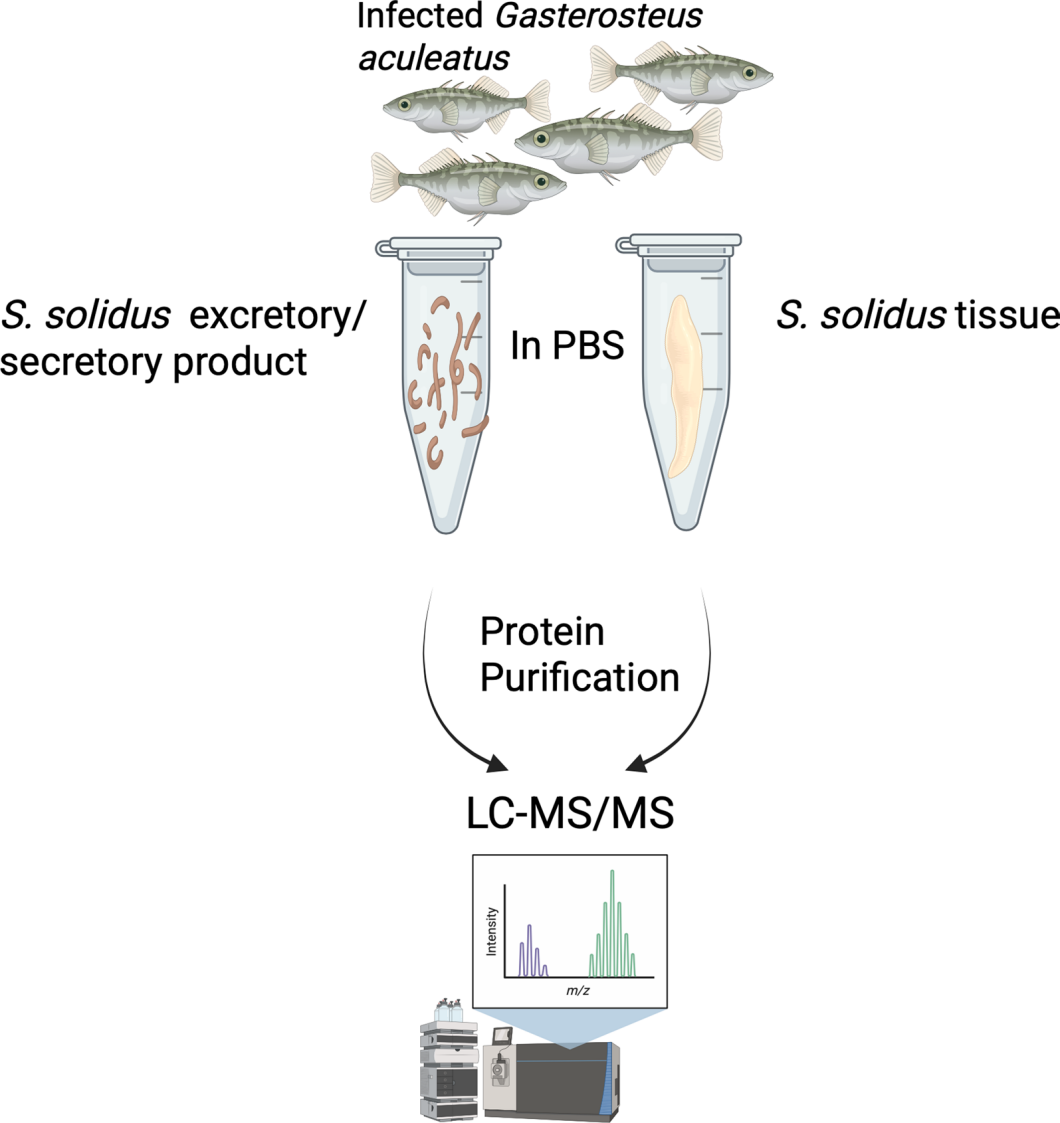
Graphical illustration of the approach for separating the ESP and whole-body tissue proteins for studying the *S. solidus* proteome. Infected three-spine stickleback is first caught, and then each *S. solidus* tapeworm is placed in phosphate-buffered saline (PBS) for 2 h before ESP. Tissue proteins are then extracted and purified separately for liquid chromatography–tandem mass spectrometry (LC–MS/MS) analysis. Created with BioRender (https://BioRender.com/dcgpav4)

We caught threespine stickleback from three lakes on Vancouver Island (Boot, Nimpkish, and Roselle lakes) and one Alaskan lake (Walby Lake; geographic coordinates listed in Supplementary Table S1, sampling methods Fig. 1; for a map see Supplementary Figure S1). The four lakes chosen as field sites are associated with information from previous years in which parasites and hosts were collected to document infection rate and parasite prevalence. Roselle and Nimpkish lakes were chosen as they are geographically close to each other, and we suspected that the tapeworm proteome from these two sites would be similar since genomic data show the genomes of tapeworms from these two lakes to be very similar [83]. We chose Boot Lake as it is about 200 km away from Roselle and Nimpkish lakes, and prior research has shown that heavy parasitism causes fish from Boot Lake to establish an aggressive fibrotic-like immune response (Bolnick, pers. obs). Walby Lake was chosen as a collection site in Alaska, farthest away from the Vancouver Island lakes (Figure S1), so we expected to see an especially distinctive proteome from Walby Lake tapeworms.

Fish were collected in unbaited minnow traps. Sampling was conducted with the approval of the British Columbia Ministry of Environment (permit NA22-679623) and the University of Connecticut Institutional Animal Care and Use Committee (IACUC; protocol no. A21-025). Fish were euthanized in MS-222 (tricaine mesylate), chilled at 3 °C, and quickly flown back to the University of Connecticut for dissection. Any live tapeworms dissected out of the fish were individually weighed and immediately rinsed with phosphate-buffered saline (PBS) before being placed in individual 2 ml tubes with 1000 ul of PBS following a protocol adopted from Berger et al. [24]. A total of 27 tapeworms were collected, five from fish with only a single *S. solidus* individual and 22 from fish with multiple *S. solidus* individuals (Table S2). Thirty fish from each lake were dissected to find enough tapeworms, but only two were found from Nimpkish Lake fish. Due to the unpredictability of infection rates in the collected fish, sampling of *S. solidus* from each lake did not yield equal sample sizes across lakes. Each tube was covered with aluminum foil to protect each tapeworm from light exposure that could alter the proteome composition under novel environments and to mimic the dimness of the fish peritoneal cavity where plerocercoid larvae of *S. solidus* are typically found. This technique was developed according to protocols from Berger et al. [24]. Harvesting ESPs while *S. solidus* is still in the host is difficult, since such a method would not yield enough protein to be collected for further analysis. Thus, collecting ESPs in an environment that mimics the body cavity of the fish works best even if this may lead to different results than collecting ESPs from a live fish. A protease inhibitor was not added to preserve proteins in the secretome as the tapeworms release proteins, because previous studies have shown this induces changes to the proteome composition [34]. After 2 h in the tube, the tapeworm (for whole-body tissue) and PBS (for ESPs) were separately collected, flash-frozen, and stored at −80 C° for proteomic analysis.

For each tapeworm whole-body tissue sample, tissue was cut and placed in a tube containing lysis buffer, sodium dodecyl sulfate with Tris, and dithiothreitol. The use of whole-body tissue as a baseline was to confirm whether ESPs were uniquely observed or also present in the proteome of *S. solidus*, ensuring that observed differences in ESPs reflected true secretory or excretory activity rather than residual tissue contamination. The scolex and strobilar portions of the tapeworm were both used in sample preparation. A mixture of sterile ceramic and glass beads was added to each tube, and samples were homogenized for 1 min at 5000 rpm using a Fisherbrand bead mill homogenizer (Fisher Scientific). Homogenization was repeated for three cycles, with samples placed on ice between each cycle to prevent overheating. Samples were then centrifuged at 8000×*g* for 2 min, and the supernatant of each sample was transferred to new tubes. Protein concentrations were measured on a NanoDrop spectrophotometer (A280 nm; Thermo Scientific) before proceeding to the protein digestion protocol. For secretome samples collected in PBS, protein concentrations were directly measured before proceeding to the same digestion protocol.

### Untargeted protein identification and label-free quantification via tandem mass spectrometry

#### In-solution digestion protocol for protein purification

For reduction/alkylation of Cys residues, 200 mM of ammonium bicarbonate was added to each sample at a 1:1 sample/buffer ratio to make a working concentration of 100 mM (pH 8). A total of 100 mM of fresh dithiothreitol (6 ul for each sample) was then added to yield a final sample concentration of 5 mM (pH 8), and then reduced for 90 min at room temperature on a shaker. The sample was then alkylated in the dark with 100 mM of fresh iodoacetamide (11 ul for each sample) for 45 min at room temperature on a shaker. Once samples were alkylated, proteolysis was conducted using a modified porcine trypsin protease (Promega #V5113) added at a ratio of 1:20 w/w enzyme/protein at 37 °C and incubated overnight. The next day, samples were acidified with concentrated formic acid (pH 3).

Samples were then transferred to a Pierce peptide desalting high-capacity spin column (Thermo Fisher #89852) and desalted by reversed-phase chromatography. To prepare spin columns for desalting, columns were first centrifuged at 5000×*g*, then washed with pure acetonitrile twice before a final washing with buffer A (0.1% formic acid diluted in Fisher Optima LC/MS-grade water). Acidified samples were loaded into each spin column and washed twice with buffer A, eluted twice with an elution buffer (50% acetonitrile in 0.1% formic acid), and finally allowed to dry completely before resuspension in buffer A (0.1% formic acid in Fisher Optima LC/MS-grade water). Samples were quantified by A280 absorbance, and total concentrations were normalized.

### Liquid chromatography–mass spectrometry (LC–MS) analysis

*Schistocephalus solidus* samples (500 ng each) were analyzed using a Thermo Scientific UltiMate 3000 RSLCnano ultrahigh-performance LC (UPLC) system coupled to a high-resolution Thermo Scientific Q Exactive HF mass spectrometer. Each sample was injected onto a PepMap RSLC C18 column (2 μm, 75 μm × 25 cm, Thermo Fisher #ES902) and separated by reversed-phase UPLC using a gradient of 4–30% solvent B (0.1% formic acid in Fisher Optima LC/MS-grade acetonitrile) over a 50-min gradient and 30–90% solvent B over 10 min, followed by column washing and re-equilibration at 300 nl/min flow and 50 °C. Peptides were eluted directly into the Q Exactive HF using positive-mode nanoflow electrospray ionization. MS1 scans were acquired at 60,000 resolution, with an automatic gain control (AGC) target of 1 × 10^6^, maximum ion time of 60 ms, and a scan range of 300–1800 m/z. Data-dependent MS2 scans were acquired at 15,000 resolution, with an AGC target of 1 × 10^5^, maximum ion time of 40 ms, isolation window of 2.0 m/z, loop count of 15, normalized collision energy of 27, dynamic exclusion window of 30 s, and charge exclusion “on” for all unassigned, +1, and > +8 charged species.

### Data processing

Peptides from all samples were identified using MaxQuant software (v1.6.10.43) and its embedded Andromeda search engine, and quantified using label-free quantification [35]. Raw data from the samples were searched against the UniProt *S. solidus* proteome (identifier UP000275846, accessed 03/27/2022), the *Caenorhabditis elegans* reference proteome (UP000001940, accessed 12/15/22), and the MaxQuant contaminants database. The minimum peptide length was set to five residues for the search. Variable modifications allowed oxidation of Met, acetylation of protein N-termini, deamidation of Asn/Gln, and peptide N-terminal Gln-to-pyroGlu conversion. Carbamidomethylation of Cys was set as a fixed modification. Protease specificity was set to trypsin/P with a maximum of two missed cleavages. All results were filtered to a 1% false discovery rate at the peptide and protein levels using the target–decoy approach; all other parameters were kept at default values. MaxQuant output files were imported into Scaffold (v5.1.2, Proteome Software, Inc.) for data visualization and subsequent analyses.

We created a more robust plerocercoid proteome dataset by not filtering out small fragments of proteins and peptides, as well as by including tapeworms with mass less than 50 mg, a mass threshold used by some to define the parasite infective stage that begins to alter behavior in fish hosts [76, 77].

Proteins identified with two unique peptides were considered accurately identified, and any protein with one unique peptide was discarded. Protein transmembrane regions were predicted with DeepTMHMM (version 1.0.24, [37]) and Phobius [38]. Secreted proteins were predicted using SignalP 6.0 (using “Eukaryota” option, [79]). Protein annotation was determined from BlastP on the UniProt database with default parameters. A matrix of total spectrum counts was exported and analyzed in R (version 4.4.0) to generate non-metric multidimensional scaling (NMDS) axis scores for each sample [36]. We used a permutational analysis of variance (PERMANOVA) to test for between-group differences in the spectrum counts, either comparing tissue types (ESP vs. whole-body proteomes) or comparing among populations of tapeworms, and testing for population-by-tissue interaction effects. To identify specific proteins underlying population and tissue differences, we iterated through each protein. For each protein we used a binomial general linear model to test whether normalized total spectrum counts varied as a function of tissue type, population, or a tissue × population interaction effect. We used a strict Bonferroni correction for multiple comparisons to determine statistical significance thresholds. All data and code required for analyses and graphics presented here are available on FigShare (https://doi.org/10.6084/m9.figshare.25718403.v1).

## Results and discussion

### Composition of the *S. solidus* proteome

A total of 1362 proteins were identified from tapeworm whole-body tissue and ESPs, with 439 uncharacterized proteins (full list on FigShare: https://doi.org/10.6084/m9.figshare.25718403.v1). Proteins identified ranged from 651 DA to 5 kDA in weight; anything lighter in molecular weight was not detected. There was substantial overlap between the ESP and whole-body proteome. For example, in Boot Lake tapeworms, the ESP yielded 1319 proteins compared to 803 from the whole body, 737 of which were shared. With a few exceptions of proteins mostly confined to the whole body, there is a tendency for proteins that are more abundant in the whole body to also be more abundant in ESPs (Supplementary Figure S2). The smaller total count of whole-body proteins may reflect the dominance of a few abundant structural proteins, leading to under-sampling of relatively rare proteins which are then identified only in the ESP samples.

Of the 1362 total proteins, 60 were predicted to have at least one signaling peptide region, 219 were predicted to have at least one transmembrane region, and 16 had both a signaling and transmembrane region (Table 1). Within the context of parasite proteins, the presence of both types of proteins can provide valuable insights into localization and function within a host organism. For example, detection of parasite signal peptides could indicate proteins that are secreted to play roles in modulating host immune responses or host cell functions. In this regard, we found that alkaline phosphatase (A0A183SJK6) contained both a signal peptide and transmembrane domain, which has been noted to be expressed in schistosomes at the host–parasite interface and has been shown to decrease host immune responses against the parasitic platyhelminths by generating immunosuppressants like adenosine to enhance parasite survival [82].

**Table 1:** Proteins detected within the *S. solidus* proteome containing both signaling peptides and transmembrane domains

Protein ID (UniProt)	Protein name	Secreted signal site (amino acids)	Transmembrane site (amino acids)	References	Other parasite species
A0A183SJ61	Perlecan (basement membrane-specific heparan sulfate	1–41	1106–1127	Frevert et al. [61], Bambino-Medeiros et al. [62]	*Plasmodium berghei*, *Trypanosoma cruzi*
A0A183T9I5	Cysteine-rich venom protein Mr30	1–21	275–297	Wilbers et al., [43]	*Trichinella spiralis*, *Strongyloides ratti*
A0A3P7CDD7	Alkaline phosphatase	1–32	520–537	Stettler et al. [63], Neves et al. [64]	*Echinococcus multilocularis*, *Trypanosoma cruzi*
A0A183SHS7	Bravo_FIGEY domain-containing protein	1–22	769–792	N/A	
A0A183TL89	Cytochrome c domain-containing protein	1–42	20–42, 246–264	Espino-Sanchez et al. [65]	*Plasmodium falciparum*
A0A183SU34	Fn3_like domain-containing protein	1–30	7–26	N/A	
A0A183T9K4	Integrin beta	1–19	523–544	Beckmann et al. [66], Figueria et al. [67]	*Schistosoma mansoni*, *Leishmania amazonensis*
A0A183SWB9	Alpha-carbonic anhydrase domain-containing protein	1–21	228–248	Angeli et al. [68], Da’dara et al. [69]	*S. mansoni*
A0A183SHE2	Zinc transporter ZIP10	1–29	91–116, 128–150, 170–191, 412–432, 438–456, 477–497	Schulte et al. [70]	*Schistosoma japonicum*, *P. falciparum*
A0A0X3PMB9	SLEEPLESS protein	1–26	127–144	N/A	
A0A183T717	GOLD domain-containing protein	1–23	185–204	Yang et al. [71]	* Plasmodiophora brassicae*
A0A0X3PGH0	Leucine-rich repeat protein	1–19	552–575	Kedzieski et al. [72], Freville et al. [73]	*Leishmania infantum*, *Leishmania major*
A0A183SE30	Integrin_alpha2 domain-containing protein	1–26	1410–1434	Chesnokov et al. [74]	*P. falciparum*
A0A183TQE2	Peptidase_M28 domain-containing protein	1–17	378–400, 412–434, 454–476, 517–535, 541–561, 573–598, 610–633, 645–669	Escotte-Binet et al. [75], Bos et al. [76]	*Toxoplasma gondii*, S.* mansoni*
A0A0V0J548 A0A3P7CVC8	Ig-like domain-containing protein Uncharacterized protein	1–18 1–23	154–177 130–155	N/A N/A	

**Fig. 2:**
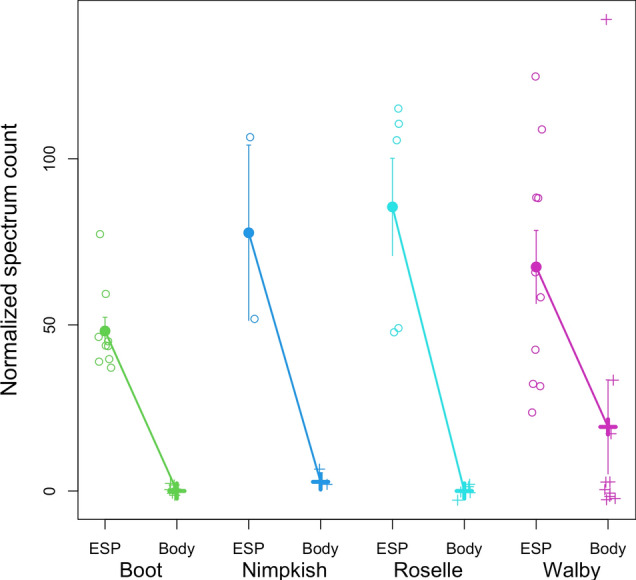
Normalized spectrum count of paramyosin. ESP stands for excretory/secretory protein, and body indicates paramyosin found in the tissue proteome. Raw observations are plotted in open circles, filled points are means with one-standard-error confidence intervals for each tissue within each population. A general linear model confirms that paramyosin exhibits statistically significant differences between ESP and tissue (*P* < 0.0001) and among populations (*P* = 0.0159), and exhibits a population × tissue interaction (*P* < 0.0001), indicating that the ESP–tissue difference is larger in some lakes than others

Signaling peptides are responsible for directing proteins to their appropriate cellular location, which can include functions like secretion out of a cell or targeting specific organelles within a cell. We identified 15 proteins with domains indicating they are secreted signal peptides which were abundantly found in the secretome from all four lakes. Eight of these proteins have not been previously reported in *S. solidus* literature, which may be attributed to geographical differences between the lakes sampled here and those in prior studies, as such variation can naturally lead to differing results (Table 2). Two of the newly detected secretome proteins (A0A3P7EX19 and A0A3P7EFK9) resemble immunoglobulin domain proteins which may have immunoregulatory properties in regulating stickleback infections, but the proteome is not annotated well enough to suggest what those may be. Additionally, five of these secretory proteins belong to either the large (60S) or small (40S) ribosomal subunit family (A0A0V0J1I4, A0A183TKA8, A0A0V0J8X3, A0A183TM78, A0A3P7BWG1), responsible for ribosome synthesis and ribosomal RNA (rRNA) processing. We also identified paramyosin (A0A3P7DV89), a myofibrillar protein that has been shown in other helminth parasites to play a multifunctional role in host–parasite interactions (Fig. 2) [39–41]. Across all lake populations, paramyosin had an 8.8-fold higher total spectrum count in the ESP than in the whole-body tissue (*P* < 0.0001). Paramyosin is a rod-shaped α-helical myosin-binding protein commonly found in invertebrates through linking with actin filaments and tropomyosin, most likely involved in specialized contractile functions [42]. Paramyosin in *Clonorchis sinensis*, *Schistosoma mansoni*, and *Trichinella spiralis* has been shown to bind and stimulate host complement component proteins, which allows the parasite to evade the host’s innate immune system [39, 40, 42]. Previous work using the *G. aculeatus* and *S. solidus* model demonstrated that *S. solidus* infection modulates the innate immune system, with complement and T regulatory responses upregulated in hosts infected with high-growth *S. solidus*, emphasizing the role of immune timing and coevolutionary dynamics in host–parasite interactions [80].

**Table 2:** Secreted signal peptides common to the secretome of *S. solidus* from all four lakes

Protein ID (UniProt)	Protein name	Secreted signal peptide/transmembrane sites	UniProt/InterPro annotation	Reported in Kochneva et al. [25]	Reported in Berger et al. [24]
A0A183SYT6	Reverse transcriptase domain protein	None	Transcriptase	No	Yes, detected in secretome and proteome
A0A183T614	Calcium-transporting ATPase	Four transmembrane sites	ATP binding, P-type calcium transporter activity	No	Yes, detected in secretome and proteome
A0A3P7EX19	Ig-like domain-containing protein	None	None	No	No
A0A183T1U1	ATP-dependent 6-phosphofructokinase	None	ATP binding, metal ion binding	No	No
A0A3P7EFK9	Ig-like domain-containing protein	Two transmembrane sites	Cellular membrane component	No	No
A0A183T1A6	Succinate dehydrogenase [ubiquinone] iron-sulfur subunit, mitochondrial	One signal site	Metal ion binding, electron transfer activity, iron sulfur cluster binding	No	No
A0A3P7CSE3	Major vault protein	One signal site	Ribonucleoprotein complex	No	No
A0A3P7DV89	Paramyosin	None	Myosin complex	No	No
A0A0V0J1I4	60S acidic ribosomal protein P0	None	Ribosome biogenesis	No	Yes, only detected in proteome
A0A183TKA8	60S ribosomal protein L3	None	Translation, structural constituent of ribosome	No	No
A0A0V0J8X3	Small ribosomal subunit protein uS5	None	RNA binding, translation	No	Yes, detected in secretome and proteome
A0A183TM78	Small ribosomal subunit protein uS2	None	Structural constituent of ribosome, translation	No	Yes, detected in secretome and proteome
A0A3P7BWG1	40S ribosomal protein S4	None	rRNA binding, translation	No	No
A0A183SXP6	Sidoreflexin	Three transmembrane sites	Monatomic ion transmembrane transporter activity	No	Yes
A0A183SWW7	Innexin	Nine transmembrane sites	Monatomic ion transmembrane transport	No	Yes, detected in secretome and proteome

Transmembrane proteins are components of cellular membranes and perform functions such as cell signaling, cell–cell communication, or transport of molecules across membranes. Parasite transmembrane proteins can interact with host cell membranes, which may lead to evasion of host defense mechanisms. Among the 16 identified proteins that had both a signaling peptide and transmembrane region, 11 were found in other parasites with known functions in maintaining host–parasite interactions, and six in helminth parasites (Table 1). One protein (A0A183T9I5) is a secreted venom allergen-like (VAL) protein that is structurally conserved and abundantly secreted in multiple stages of helminth parasites [43]. VALs were initially studied for their immunogenic properties and potential as vaccine candidates due to their role in protective immunity. In animals, parasites produce VALs from secretory glands and modulate host immune responses through inhibition of platelet aggregation, granulocyte adhesion, oxidative burst, and B cell signaling pathways [43–46].

### Multivariate analyses of proteome divergence among tissues and among populations

**Fig. 3:**
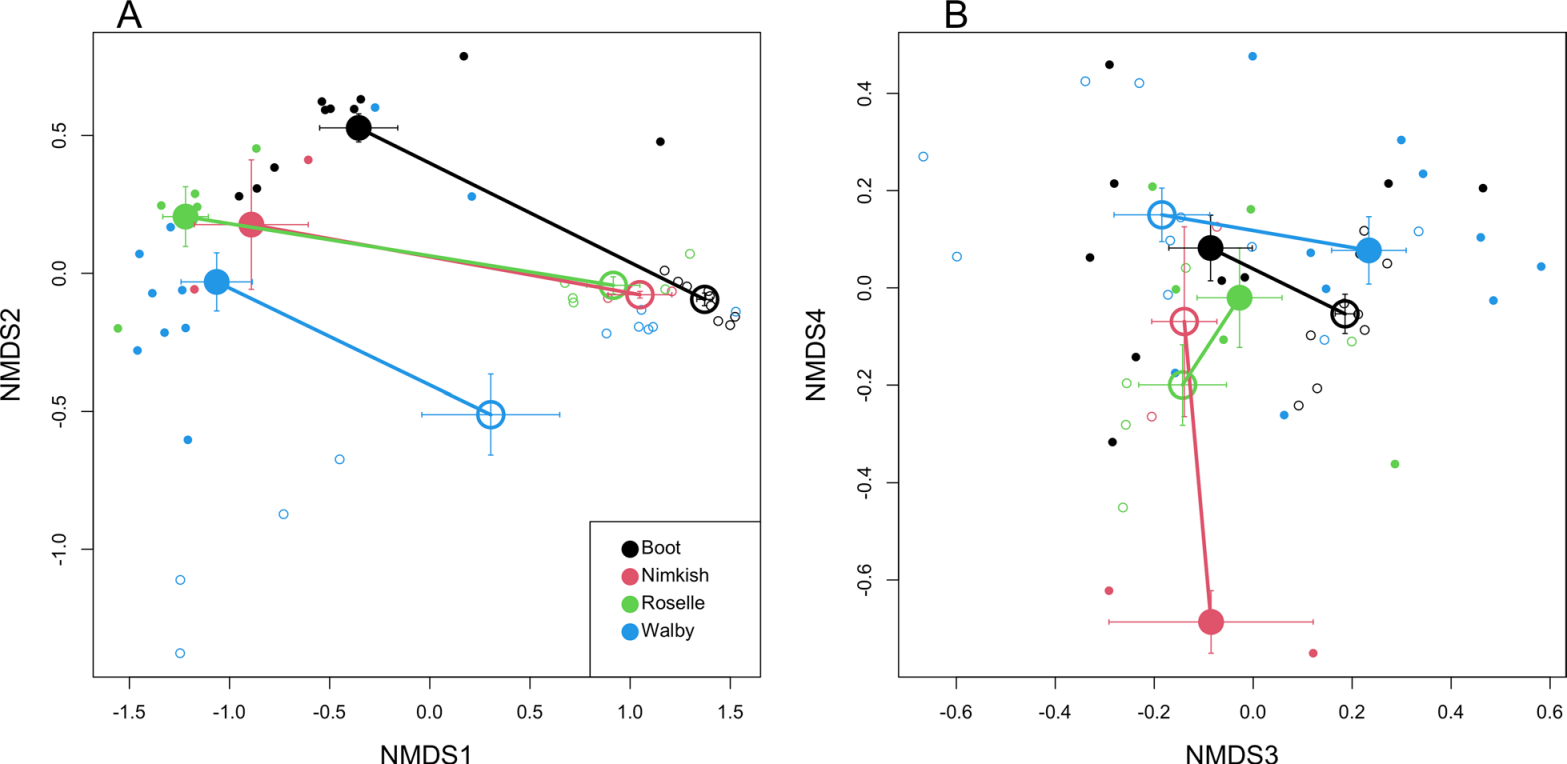
Lake origin and differences in the ESP and tissue proteome were examined. Lake origin affects differences in proteome less than ESP vs. tissue proteins. We used the normalized spectrum count from each protein found in each fish to calculate NMDS scores. Lake origin is color-coded with a larger circle and symbol to denote standard error bars. The closed and open circles represent tissue and ESP proteins, respectively. **A** NMDS 1 and 2 of ESP and tissue proteome. **B** NMDS 3 and 4 of the entire proteome combined for all lakes

A major goal of this study was to evaluate whether the proteome differed among parasite populations. To compare the ESP and whole-body tissue proteome across the four lakes, we performed a multivariate analysis (NMDS with *K* = 8) to differentiate all eight groups (four lake populations by two sample types), in a single summary analysis of the whole proteome data matrix. The NMDS axes represent major combinations of proteins that best differentiate samples into the eight groups. Loadings of proteins on the first four NMDS axes are provided in Supplementary Table S3 and represent the importance of each protein in defining a given NMDS axis. To test the statistical significance of these differences, a multivariate ANOVA (MANOVA) applied to NMDS axes 1–4 confirmed a significant effect of tissue type (Pillai’s trace = 0.87, *F*_2,50_ = 172.42, *P* < 0.0001) and genotype of the parasite (Pillai’s trace = 0.908, *F*_12,123_ = 4.45, *P* < 0.0001), and genotype-by-tissue interaction (Pillai’s trace = 0.674, *F*_12,132_ = 2.97, *P* = 0.0011). The significant genotype-by-tissue interaction indicates that the difference between the proteins in the body and ESP depends on the parasite population. The first NMDS axis (Fig. 3) separates ESP versus whole-body tissue proteome for all four populations. Both the first and second axes separate the populations, but the third and fourth NMDS axes exhibit population-specific differences between ESP and whole-body tissue. These population-specific differences are strong candidates for proteins involved in local adaptation to their local host populations.

We also observed statistically significant differences only within the ESP proteome between the different tapeworm populations (Pillai’s trace = 1.19, *F*_12,63_ = 3.44, *P* = 0.0006; Supplementary Table S4). Similarly, we observed statistically significant differences in the tapeworm whole-body tissue proteome composition between tapeworm populations (Pillai’s trace = 1.68, *F*_12,66_ = 6.58,* P* < 0.0001, Supplementary Table S4). Among-population variance in ESP composition is not significantly greater than among-population variance in whole-body proteomes (*F*-test of the ratio of variances, *F*_4,4_ = 1.285, *P* = 0.2830). This suggests that both ESP and whole-body tissue proteomes are diverging among populations, and the ESP proteins are not uniquely important for population adaptation or coevolution.

Although we observe statistically significant differences between populations, it is important to acknowledge that there are also important similarities. Most proteins identified in the ESP samples were shared among all populations’ ESP samples. For instance, we identified 1319 proteins in Boot Lake ESP and 832 in Nimpkish Lake ESPs, 826 of which were found in both populations. Nimpkish Lake and Roselle Lake body samples had 180 and 184 proteins identified, respectively, 113 of which were shared. For more details on other pair-wise overlaps, see Supplemental Figure S3. Going beyond mere overlap, there were strong positive correlations between protein abundance in different populations. Depending on the pair of lakes being compared, these correlations ranged from 0.702 to 0.876 (whole body) and from 0.753 to 0.992 (ESP), all of which are statistically significant at *P* < 0.001 (Supplemental Figure S3). Of particular note, the geographically closest pair, Roselle and Nimpkish lakes, had the highest correlations between their ESP proteins, and the geographically most distant Walby Lake ESP had the lowest correlations with other lakes’ ESPs.

### Among-population differences in abundance of particular proteins

Individual protein analyses revealed many that differed significantly between tissues and between populations (Supplementary Table S5). We identified a total of 186 proteins whose spectrum counts differed among the four lakes, using a strict Bonferroni false discovery rate correction. Of these 186 proteins, 60 were uncharacterized. Of the characterized proteins, 67 can be assigned to 21 protein families. Tubulin was the largest family, with seven tubulin alpha chain proteins (A0A183SBQ0, A0A183SL33, A0A183T3L3, A0A183T3T2, A0A183TBY5, A0A183TD95, A0A183TJU1) and six tubulin beta chain proteins (A0A183SFW7, A0A3P7DQX4, A0A3P7D6K9, A0A3P7D9Q4, A0A0X3NM14, A0A0X3PFM5). Tubulin alpha proteins consistently had higher spectrum counts in the tissue of tapeworms compared to their ESP in three lakes (Boot, Roselle, and Walby, all *P* < 0.0001). However, in Nimpkish Lake, this protein was enriched in the ESP compared to tissue (*P* < 0.0001). Tubulin beta chain protein had higher spectrum counts in the ESP than in tapeworm tissue in Nimpkish Lake and Walby Lake only (both *P* < 0.0001). Spectrum counts were higher in tubulin beta than tubulin alpha because beta sheets typically contain 42% of tubulin beta and 39% of tubulin alpha [60]. Tubulin alpha chain proteins are almost always detyrosinated, which makes them susceptible to faster degradation than their tyrosinated counterparts.

The second-largest family that differed among lake populations was dynein, with seven dynein light chain proteins (A0A0V0JBQ8, A0A183SE96, A0A183SE97, A0A183SKR1, A0A183TQI2, A0A183TQX4, A0A0X3P671). Of note, A0A183SKR1 had the highest spectrum count only in the whole-body tissue of tapeworms from Boot Lake (*P* < 0.0001); it was nearly absent in all ESPs and whole-body tissues from all other lakes. Additionally, three paramyosin proteins (A0A3P7BUS1, A0A183T4B5, A0A3P7CMF5) exhibited higher spectrum counts (*P* < 0.0001) in the ESP of all tapeworms than in tissue samples. Specifically, A0A3P7BUS1 averaged less than 10 spectrum counts, while the others had at least 50 counts, with an average of around 100 counts.

These differences in protein expression across lake populations may suggest that local environmental conditions (including host traits) are driving distinct molecular adaptations in tapeworms. For example, enrichment of tubulin alpha chain proteins in tapeworm tissues in all lakes except Nimpkish Lake could reflect variations in the influence of environmental stressors such as temperature, nutrient availability, or host immune responses on cytoskeletal dynamics. Higher abundance of tubulin beta chain proteins in the ESP of Nimpkish and Walby lakes may indicate a role in extracellular signaling or structural stability under ecological pressures. Alternatively, tapeworms found in Nimpkish Lake were lighter in mass than tapeworms found in other lakes, and given the degradation rate of detyrosinated tubulin alpha proteins, mass may be a contributing factor to the differential expression of these proteins.

We found a cluster of six actin proteins (A0A183SVY0, A0A183T1Z7, A0A0X3PDD0, A0A183TJY3, A0A183S955, A0A183TGM4) that were significantly different in the ESP and tapeworm whole-body tissue (all *P* < 0.0001), as well as significantly different in the level of actin abundance within tapeworm tissue among the different lakes (Supplementary Figure S4). Two paramyosin proteins (A0A183T4B5, A0A3P7CMF5) appear to be differentially abundant across different lakes (*P* < 0.0001) as well as between the ESP and whole-body tissue proteome (*P* < 0.0001) (e.g., Fig. 2). Tapeworm whole-body tissue either did not contain either protein or had a spectrum count of less than 10. Both proteins have spectrum counts above 100 in the ESP of all tapeworms except for six from Walby Lake, with total spectra highest in Roselle Lake ESP. Other muscle proteins involved in muscle formation like transgelin (A0A183SYR3, Figure S6), tubulin (A0A183TBY5, Figure S7), and myosin (A0A183T9Q5) were found to have higher spectrum counts in the ESP than tissue proteome (all *P* < 0.0001), with the tissue proteome from Nimpkish, Roselle, and Walby lakes typically showing two spectrum counts or less. Immunohistochemical studies in other tapeworms such as *Echinococcus granulosus* and *Taenia solium* have shown these myofibrillar proteins to bind to host Fc fragments on immunoglobulins, complement C1, and complement C9, making them attractive targets for parasite diagnostic tests and pharmacological development [47].

Aside from muscle proteins, we additionally identified two HSPs, HSP70 (protein ID:A0A183S9K7) and HSP90 (protein ID: A0A183SJF6), with total spectrum counts highest in the ESP of tapeworms found in Boot Lake, but lowest in ESP of tapeworms in Walby Lake (tissue difference *P* < 0.0001, population effect *P* = 0.0003, and tissue-by-population interaction effect *P* = 0.0008). This is representative of several proteins whose body/ESP difference varies significantly among lakes (significant population × tissue interactions, Supplementary Table S4). HSPs are a family of highly conserved molecular chaperones found in eukaryotic organisms and are important in cellular processes, helping denatured proteins to refold or targeting them for degradation [48]. HSPs are expressed under normal conditions, but expression increases under stresses such as temperature changes, heavy metal exposure, and parasite infections. HSPs are grouped based on their molecular weight, so major groups are typically denoted under HSP100, HSP90,HSP70, HSP60, HSP40, and smaller HSPs. During parasite development, transmission through different hosts during developmental stages, like passing through cold-blooded hosts such as fish, mosquitos, and insects and switching to homeothermic hosts, requires HSPs to regulate for the change in such drastically different environments. HSPs are typically present to maintain homeostasis by functioning as molecular chaperones; thus, their presence in the ESP is intriguing. It has been reported that some HSPs function as moonlighting proteins, performing multiple roles within a single polypeptide chain [81]. These proteins exhibit multiple functions not due to gene fusions or having multiple proteolytic fragments, but through interaction with a targeted cell surface or when they are secreted.

HSPs and their role in modulating helminths’ response to their environments have been reported in several species [48, 50]. HSP70 in *Echinococcus multilocularis* is found to be released as extracellular vesicles by the protoscoleces, which could potentially promote angiogenesis [49]. The C-terminal region of HSP70 in *E. granulosus* has been shown to induce host immunoglobulin G (IgG) and IgE response [50]. *Clonorchis sinensis* produces both HSP70 and HSP90 in its adult stage, with both proteins inducing a TH1 response in hosts [52]. HSP90 has been found in extracellular vesicles in various helminths that can regulate host macrophage functions and modulate other immune responses [51, 53].

These examples highlight a broader trend for the relative abundance of many proteins to differ among tapeworm populations. The causes of these among-population differences have yet to be established: it is important to consider both intrinsic genetic variation (gene expression rate, translation rate, protein stability, post-translational modifications) and extrinsic environmental effects (e.g., ambient temperature, host immune response, host genotype). Parasite proteome differences might, for example, be induced by host genetic differences that lead to population differences in host immune response. Future studies should expand on these results by experimentally rearing different parasite genotypes in different host genotypes and across different abiotic conditions. The observed patterns may provide insights into molecular mechanisms underlying adaptation but also raise questions about the functional consequences of these differences for both tapeworm survival and host–parasite dynamics.

### Comparison with prior studies of *Schistocephalus* secretomes

In a prior study of *S. solidus* from Quebec, eight proteins were reported to be found exclusively in the secretome of all sampled individuals [24]. None of these were detected in our samples. In that prior proteome study, an additional 22 exclusive secretome proteins were detected only in some of the proteome samples [24]. However, only three of these proteins (A0A183TPG4, A0A183TIR8, A0A183TE24) were identified in our dataset, but none were secretome specific. We attribute these discrepancies to differences in geographical location as well as how the threespine stickleback in the prior study were raised prior to each experiment or bioinformatics methods employed for analysis [24]. A0A183TPG4 was only found in five tapeworms from Boot Lake and one from Nimpkish Lake. However, all five tapeworms from Boot Lake had only one unique peptide, which falls below our filtering threshold. The protein contains a cystatin domain, which may have host immunomodulatory properties. Cystatin is a protease inhibitor primarily secreted by parasites to evade host immune responses [54]. A0A183TIR8 was found in at least one tapeworm from each lake. The protein functions as a sodium/glucose cotransporter protein involved in mediating sodium and glucose transport across cell membranes. Since *S. solidus* does not possess a digestive system and instead obtains nutrients by consuming carbohydrates (or glucose) through the glycolytic pathway, proteins that regulate glycolytic processes are especially important for survival. A0A183TE24 is an intraflagellar transport protein required for the maintenance and formation of cilia. We found this protein in at least one tapeworm from every lake (11 total) in both tissue and secretory samples. However, only four out of the 11 samples had a spectrum count above the minimum filtering threshold, but the molecular weight of each sample was the same across all samples, so it is likely due to the low quantity of the sample at the processing stage. The coracidia of *S. solidus* emerge from their eggs with cilia that aids in swimming. However, once ingested by a copepod, coracidia shed their cilia. Therefore, this peptide is not expected to be present once they are in their second intermediate host [55].

Within the ESP, we found several excreted proteins that have been detected in some plerocercoids of *S. solidus* tapeworms found in freshwater lakes across Russia [25]. Malate dehydrogenase (A0A183TNV9) was excreted in all tapeworms, although it had previously been reported to be found only in some *S. solidus* tapeworms [25]. We believe the discrepancy was due to varying methods related to sample preparation for mass spectrometry. Malate dehydrogenase is an enzyme that catalyzes nicotinamide adenine dinucleotide (NAD/NADH)-dependent interconversion of malate and oxaloacetate, a crucial mediator in gluconeogenesis and the citric acid cycle, and as a malate–aspartate shuttle. In helminths, this protein plays an important role in obtaining energy from glucose and overcoming oxidative stress. It has been highlighted as a potential therapeutic drug target against parasites [56].

**Fig. 4:**
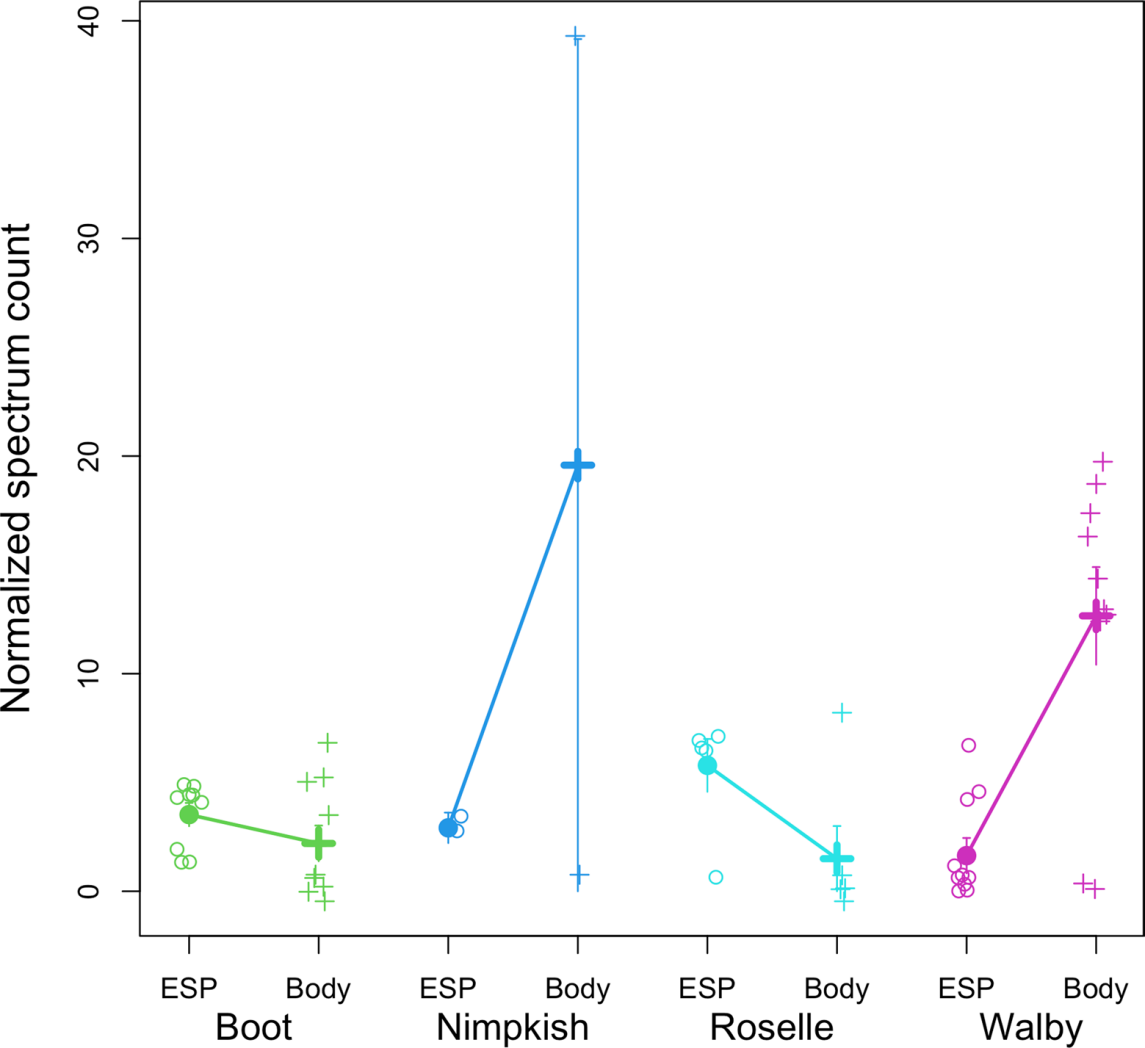
Normalized spectrum count of annexin, which shows a population-by-tissue interaction effect. Raw observations are plotted as small data points, with bold points denoting means and one-standard-error confidence intervals for each tissue within each population. A general linear model confirms that annexin exhibits statistically significant differences between ESP and whole-body tissue (*P* = 0.0160) and among populations (*P* = 0.0190), and a population-by- tissue interaction (*P* < 0.0001), indicating that the ESP–body difference is larger in some lakes than others or reverses direction

Peptidyl-prolyl cis-trans isomerase (A0A0X3Q4H0), also known as immunophilin, was found to be excreted in all tapeworms except three plerocercoids from Walby Lake. Immunophilins are highly conserved proteins that catalyze protein folding by accelerating the cis/trans isomerization of peptide bonds and are thought to have roles in chaperone activity. In parasites, immunophilins have been of interest due to evidence that they are involved in the pathogenesis of infections and have strong inhibitory effects on certain parasites in culture and animal model infections, making them potential drug targets or mediators of drug action against parasites [58]. We also found a collagen-like protein (A0A183SRL7) that was excreted from all tapeworms except six plerocercoids from Walby Lake. It was previously thought that *S. solidus* released collagen into their hosts; however, the function is unclear. It could be a waste product excreted passively or potentially stimulating fibrillogenesis in hosts [24, 25]. These proteins were found in all lakes except Walby Lake, aligning with the hypothesis that parasites from distant lakes may exhibit more distinct proteomes. This pattern suggests that geographical distance can drive proteomic variation, potentially due to environmental differences or host–parasite coevolution in isolated ecosystems. A cluster of four annexin proteins (A0A183TKJ8, A0A183SWE1, A0A183SGJ0, A0A183S9W9) were found to be excreted in all four lake populations (Fig. 4). Annexins are a family of protein with diverse functions found in parasite structure and secretions that have recognized roles in the pathogenesis of parasite infections [57]. In parasites, annexins have been found to downregulate the immune response in their hosts, calcify parasite cysts, contribute to tissue development, and modulate inflammation [59]. Due to their multifaceted functions in pathological processes, annexins have been suggested as potential therapeutic treatment against *Leishmania*, *T. solium*, *E. granulosus*, *Toxoplasma gondii*, *Trypanosoma brucei*, and others [57, 59].

## Conclusions

In summary, we identified secreted, excreted, and transmembrane proteins crucial for host–parasite interactions in *S. solidus*. Highlighted proteins in the ESP may mediate these interactions, alongside new ESPs found in the *S. solidus* proteome. A comparison of ESPs and tissue proteins from different lake populations revealed previously undescribed differential expression levels in the *S. solidus* proteome. These findings underscore the potential for microevolutionary divergence in parasite proteomes among populations within close proximity. Overall, this proteomic resource enhances the understanding of host–parasite interactions and aids in identifying potential protein targets for facilitating studies of diverse interactions in nature.

## Supplementary Information

Supplementary Material 1.

Supplementary Material 2.

Supplementary Material 3.

Supplementary Material 4.

Supplementary Material 5.

Supplementary Material 6.

Supplementary Material 7.

## Data Availability

All data and R code to reproduce results of this study are publicly archived on FigShare at 10.6084/m9.figshare.25718403.v1.
