# Supplementary material for: Among-population proteomic differences in Schistocephalus solidus based on excretory/secretory and total body protein predictions
Source: Parasit Vectors. 2025 May 20;18:180. doi: 10.1186/s13071-025-06807-x (PMC12090676; doi:10.1186/s13071-025-06807-x)
Supplement: Supplementary file 2 — Supplementary Material 2. [file 13071_2025_6807_MOESM2_ESM.docx]

**Supplementary Figures S1-S7 for:**

**A. Wang & D.I. Bolnick, Among-population proteomic differences in *Schistocephalus solidus* based on excretory/secretory and total body protein predictions**


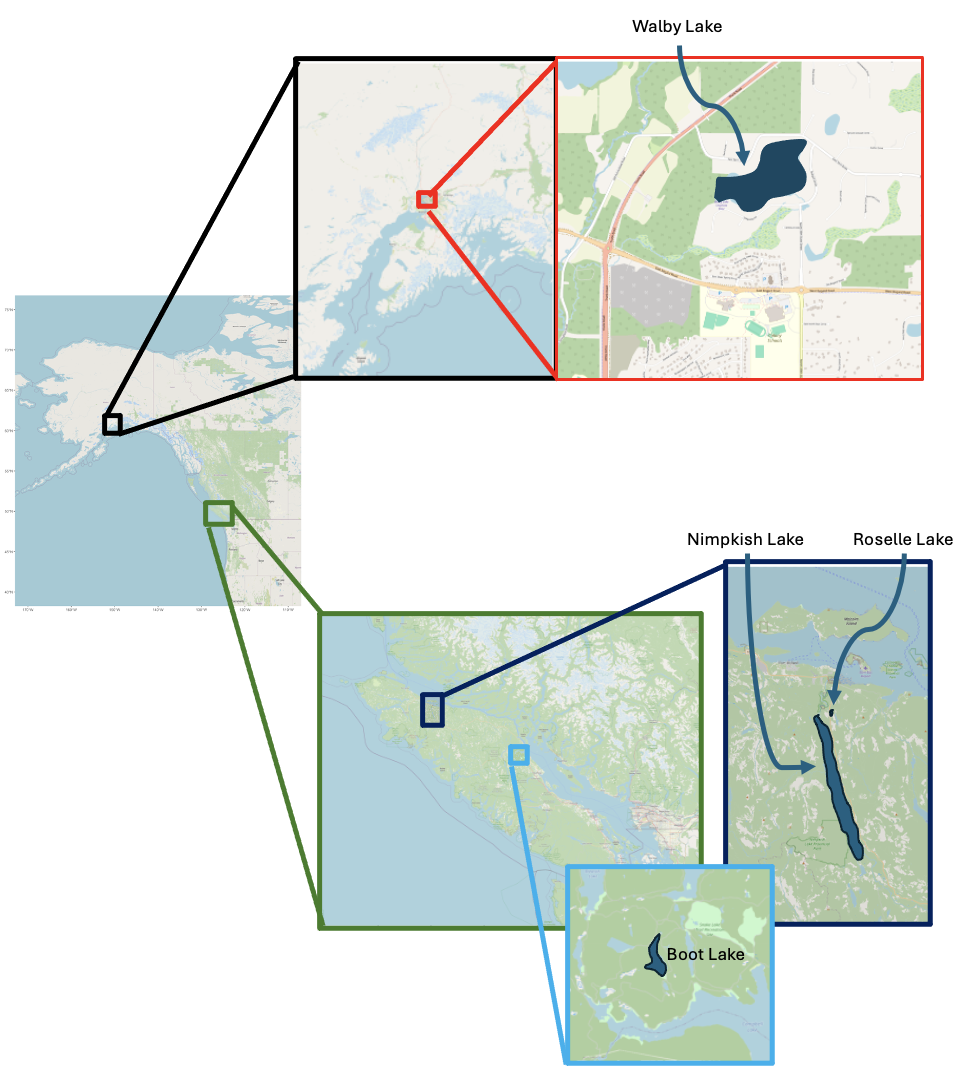


**Supplementary Figure S1.** Map of collection sites from Vancouver Island and Alaska.

**Supplementary Figure S2.** Proteomic similarity and overlap of *Schistocephalus solidus* ESP and whole body tissue. Each panel represents one of the four sampled populations (clockwise from top left, Boot Lake, Nimpkish Lake, Walby Lake, Roselle Lake). Within each panel, each point is a distinct protein type, with x and y axis values representing that protein’s abundance (normalized peak area) in whole body and ESP, respectively. Each point is the average relative abundance for a given protein across replicate individuals from that population and tissue types. The number in each axis label represents the total number of unique proteins identified for that tissue in that population. At the top of each panel we list the number of proteins shared by the two types of biological sample (the intersection of the numbers on the axis labels), and the correlation coefficient between the two tissue types relative abundances. A higher correlation indicates shared proteomic profile. Points close to an axis represent proteins that are abundant in one tissue type but not another, such as the four points with high x axis values but zero y axis values in Roselle Lake indicating proteins exclusive to the whole body.


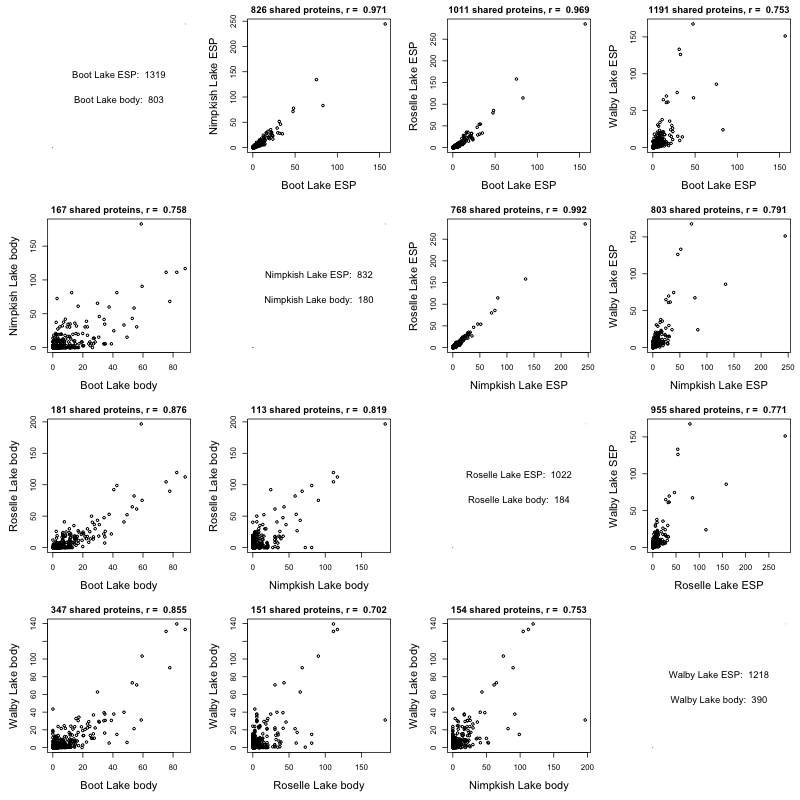


**Supplementary Figure S3.** Proteomic similarity and overlap between populations. Above the diagonal (top right) we plot results for ESP, showing the correlation between ESP protein relative abundances between each pair of populations (Boot Lake first row, Nimpkish Lake second row, etc). Each point is a protein, representing the average normalized abundance of that protein from that lake and tissue (averaged across replicate individuals). Above each panel we list the number of proteins shared between the two populations being compared, and the correlation coefficient between their proteins’ relative abundances. Walby Lake has far lower correlations with the other lakes, than the Vancouver Island lakes do with each other, consistent with its greater geographic distance. Below the diagonal (bottom left) we plot the similar results for whole body data. All correlation coefficients presented are statistically significant (P<0.0001). Along the diagonal separating the ESP and whole body panels, we print information on the total number of proteins identified in the ESP and whole body samples for each population.


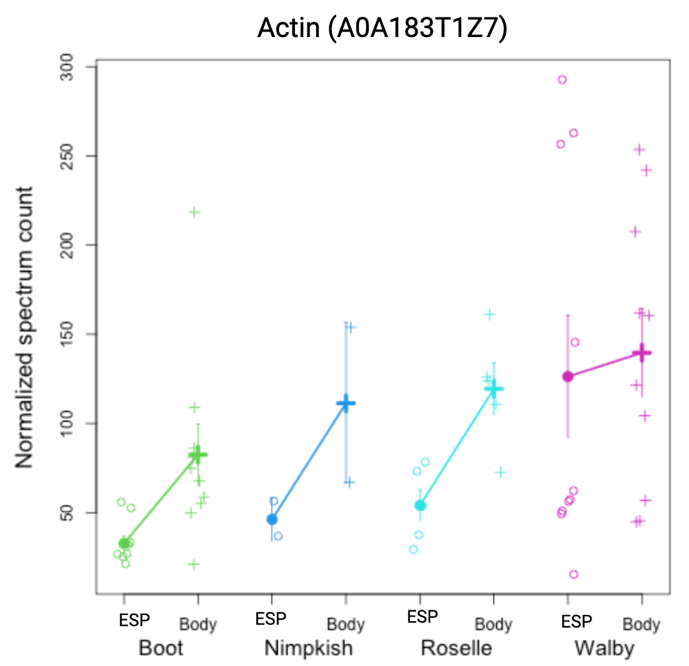


**Supplementary Figure S4.** Normalized spectrum count of actin, which shows a population by tissue interaction effect. Raw observations are plotted in open circles, with means and 1 standard error confidence intervals for each tissue within each population. A general linear model confirms that annexin exhibits statistically significant differences between SEP vs whole body (P < 0.0001), among populations (P < 0.0001), and a population*tissue interaction (P < 0.0001) indicating that the SEP-body difference is larger in some lakes than others or reverses direction.


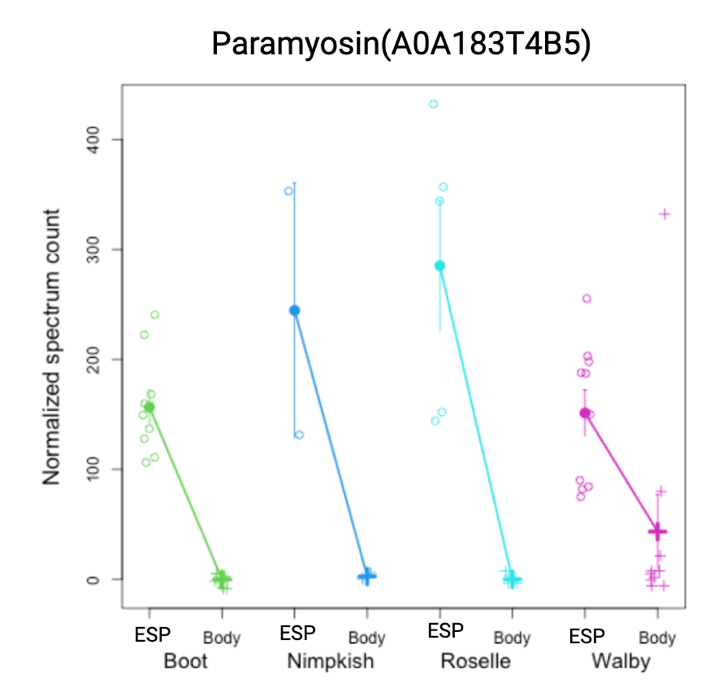


**Supplementary Figure S5.** Normalized spectrum count of paramyosin, which shows a population by tissue interaction effect. Raw observations are plotted in open circles, with means and 1 standard error confidence intervals for each tissue within each population. A general linear model confirms that annexin exhibits statistically significant differences between SEP vs whole body (P < 0.0001), among populations (P < 0.0001), and a population*tissue interaction (P < 0.0001) indicating that the SEP-body difference is larger in some lakes than others or reverses direction.


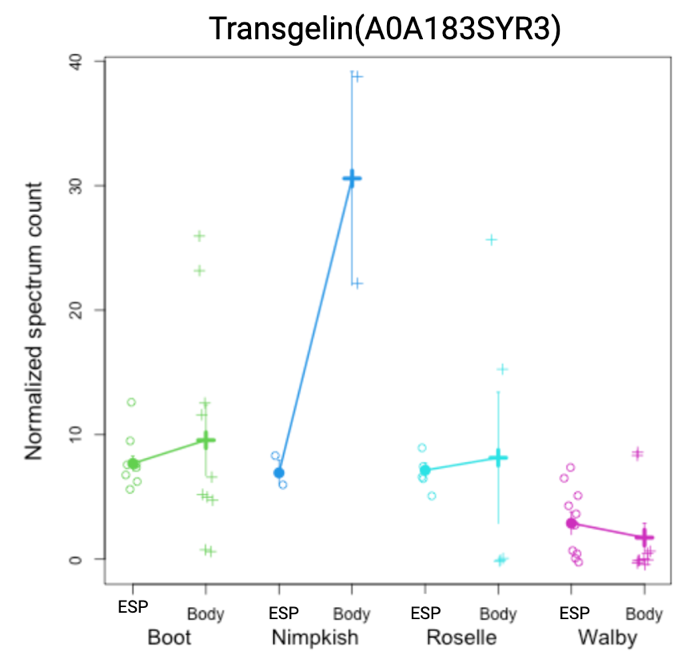


**Supplementary Figure S6.** Normalized spectrum count of transgelin, which shows a population by tissue interaction effect. Raw observations are plotted in open circles, with means and 1 standard error confidence intervals for each tissue within each population. A general linear model confirms that annexin exhibits statistically significant differences between SEP vs whole body (P = 0.216), among populations (P = 0.008), and a population*tissue interaction (P < 0.0001) indicating that the SEP-body difference is larger in some lakes than others or reverses direction.


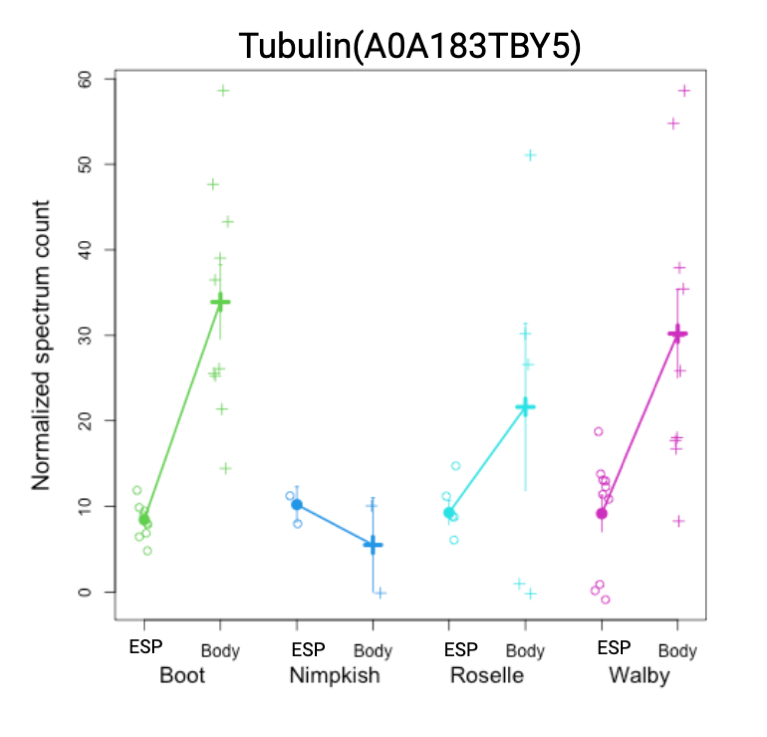


**Supplementary Figure S7.** Normalized spectrum count of tubulin, which shows a population by tissue interaction effect. Raw observations are plotted in open circles, with means and 1 standard error confidence intervals for each tissue within each population. A general linear model confirms that annexin exhibits statistically significant differences between SEP vs whole body (P < 0.0001), among populations (P = 0.995), and a population*tissue interaction (P < 0.0001) indicating that the SEP-body difference is larger in some lakes than others or reverses direction.
